# On Opacities of the Cornea

**Published:** 1850-05

**Authors:** J. C. Hall


*On Opacities af the Cornea.* By Dr. J. C. Hall.

[Dr. Hall remarks that it is very important to distinguish those opacities of the cornea which will require the assistance of art. He says:]

Opacities of the cornea, arising as they do from a great variety of causes, admit of division into three classes—

1st. Opacities resulting from the orginal structure having been destroyed by sloughing, or ulceration, followed by a deposit of opaque matter forming a cicatrix.

2nd. Opacities resulting from the application of a powerful escharotic.

3d. Cases in which opaque matter is deposited upon the texture of the cornea, without any previous loss of structure.

*Treatment of Opacities of the Cornea.*—Simple nebula of the cornea can be removed without much trouble by the daily application of a solution of nitrate of silver in distilled water—(distilled water, seven drachms; nitrate of silver, one to three grains; wine of opium, without the aromatic, one drachm.) Or what is better still, in more severe cases, by the following drops, which I have now been in the habit of prescribing very frequently during the last ten years, and with much advantage:—Take of the bichloride of mercury, two grains; distilled water, an ounce: mix for a lotion, to be used once or twice a day. I would repeat, that whatever lotion be employed, it is far better not to drop it into the eye as we commonly see, even in hospitals; a camel-hair pencil should be dipped into the solution, and the opaque spot touched with it, by which means the remedy is at once brought into direct contact with the part to which you wish its operation to be confined, and that, too, without decomposition or dilutation from the tears, which must always be the case in the ordinary method.

Whoever has had much experience in diseases of the eye, will have observed that whenever any of these drops are employed for the purpose of removing opacities of the cornea, after a few weeks they appear to lose their useful effect, the disease becoming, as it were, invincible to them ; I would, therefore, suggest that the nitrate of silver ointment, or drops, should be used for a week or ten days; then a weak solution of the bichloride of mercury; then the opium wine, and then the nitrate of silver a second time. This plan keeps up a continued state of improvement, and the disease is removed in a much shorter space of time than if one of them only had been persisted in.

When a patient comes before us with the worst possible form of opacity, leucoma arising, as is too often the case, from extensive ulceration, which has altogether changed the nature of the corneal surface, or with an extensive opacity, resulting from the application of a powerful escharotic, (which, although it may not have destroyed the vitality of the part, appears to have produced some chemical change in its structure,) it is quite labor in vain to attempt the cure of such an affection, and truly dishonest, time after time, to pick the pocket of the unfortunate patient of fees, by exciting hopes which can never be realized—hopes, the blighting of which, in the end, much increase his misery. Still, although this opacity cannot be destroyed by any means at our command, nor removed by the surgeon, this admission often only applies to the central portion of leucoma. In many cases we have the happiness to find the edges becoming gradually less and less opaque; a halo of hope surrounds this dimness of vision, and although it may not be within the compass of our art to improve the more dense central portion, something may yet be effected with the surrounding edges; and I have been consulted in cases where a steady continuance in the application of remedies has most certainly produced very great benefit, and the results recorded in the archives of the profession are doubtless such as to justify their use in many of these unfortunate cases.

According to my experience, the most useful remedy is the nitrate of silver ointment, of a strength proportioned to the condition of the affected eye. Counter-irritation should also be kept up behind the ears or at the nape of the neck by blisters. Many advise certain ointments advise certain ointments or liniments, for producing irritation on the skin. I have little practical experience of their effects, but a very short trial of them most fully convinced me that they were very inferior to blisters. In the first place, several days must elapse before any useful influence can be exerted, and, in addition to this loss of time, which is often highly importent, I am much mistaken if this St. John Long practice of applying plasters of tartarized antimony, &c., above the eyebrows, does not often produce permanently mischievous results, and too much care cannot be exercised in daily watching their effects, more particularly when applied to the head and face of young persons, in whom, in some cases, their application has been followed by a state of inflammation and sloughing which has even threatened the loss of life.

During certain stages of many inflammatory actions existing in the eye, setons and issues can be most advantageously employed. They are easily made by the surgeon, cause little pain or trouble, can be enlarged or diminished at pleasure, and being perfectly manageable, combine, within any limits we may be inclined to mark out, the advantage of a moderate degree of counter-irritation with a most salutary discharge. The advantages connected with the use of blisters are many—the convenience of their application—the rapidity of their operation—the quick subsidence of their effects when no longer required. There may, however, be cases in which, from peculiar irritability of the skin, from the liability of the patient to attacks of erysipelas, or from their having, on some former occasion, produced a violent effect on the urinary organs, some other form of counter-irritation must be used, rather than blisters.

The following is the form in which I have usually employed the nitrate of silver ointment:—To take nitrate of silver, from three to ten grains; solution of diacetate of lead, twenty drops; lard, one drachm. This ointment must be used every night or every second night. A very small portion (not larger than a large shot corn) being put into the eye. It always creates more or less ophthalmia, and its application must be regulated accordingly.

During the employment of this ointment, I would in many cases most strongly advise the internal exhibition of the bichloride of mercury. When there is nothing to counter-indicate its use, I have given it for six or eight weeks in the following form, without any severe affection of the gums, irritation of the bowels, or any other symptom requiring that the dose should either be intermitted or reduced. Ordered solution of bichloride of mercury, one drachm; tincture of bark, one drachm; distilled water, seven drachms and a half. To be taken twice a day.

As an alterative, the bichloride or oxymuriate of mercury, though doubtless more frequently prescribed than formerly in these cases, is not, I venture to think, so extensively used as it ought to be. It can be given in solu tion, which is of considerable advantage, rendering its action much more certain, more equal, and by readier absorption probably more effectual in producing an alterative influence upon the whole system.

Dr. Holland remarks that he has "seen its influence in augmenting the secretions, procuring the absorption of morbid growths, altering the state of the skin in many cutaneous disorders, and changing the character of morbid actions generally throughout the system, in cases where he believes no other medicine or combination of medicines would have had equal effect. Its combination with bark, steel, sarsaparilla, &c., afford resources of the greatest value in the treatment of disease; and, though otherwise held by common opinion, he thinks it on the whole, as safe a medicine as calomel in the hands of the practitioner, inasmuch as its distribution can be made as equal and determinate, and its effects, from being given in a state of solution, are much less likely to be interrupted by mechanical hindrances in the stomach and bowels."

I would also add, and it is worthy of note, that this medicine (bichloride of mercury) may be continued in uninterrupted use for a very considerable period, without obvious injury or inconvenience, and in certain cerebral and spinal affections, a long, unbroken course of this preparation, is of singular avail, but to reap its fullest benefits, we must be watchful, patient; and decided in its use, for in cases in which, in the end, the happiest results follow the administration of the remedy, the changes are often the slowest, and not testified by those instant and obvious results which are sometimes desirable to fortify even the mind of the physician in the perseverance proper to the practice, still more to convince the patient, his family, and friends, that time is necessary for the development of the full and complete advantages of the means employed. Thus it frequently happens that the patient, alarmed, it may be, at the name of the medicine, and by the precautions taken as to its dose and effects, or tired by the little progress he appears to make, refuses to go on with the remedy, very often at the moment when becoming most effective, and when there was every reason for thinking a dangerous or distressing malady would eventually yield by a further employment of it. I have many times seen the beneficial results arising from a long continued use of small doses of the bichloride of mercury, in solution, in chronic iritis, and also in several cases of paraplegia, the slow progress of the case giving full scope for its effects, and the great danger in prospect justifying a long trial of the remedy. Of course, in strumous children, mercury in any form can seldom be employed with advantage, and in treating opacities of the cornea in them, in addition to appropriate remedies to the eye, it will be necessary to strengthen the system by attention to diet, shower-baths, sponging the body with cold water, the administration of the disulphate of quinine, and a residence of some months during the summer, when the circumstances of the parents will permit, at the sea-side.—*London Lancet.*

